# Senolysis-Based Elimination of Chemotherapy-Induced Senescent Breast Cancer Cells by Quercetin Derivative with Blocked Hydroxy Groups

**DOI:** 10.3390/cancers14030605

**Published:** 2022-01-25

**Authors:** Anna Lewińska, Paweł Przybylski, Jagoda Adamczyk-Grochala, Dominika Błoniarz, Grzegorz Litwinienko, Maciej Wnuk

**Affiliations:** 1 Department of Biotechnology, Institute of Biology and Biotechnology, College of Natural Sciences, University of Rzeszow, Pigonia 1, 35-310 Rzeszow, Poland; jadamczyk@ur.edu.pl (J.A.-G.); dbloniarz@ur.edu.pl (D.B.); 2 Faculty of Chemistry, University of Warsaw, Pasteura 1, 02-093 Warsaw, Poland; p.przybylski5@uw.edu.pl (P.P.); litwin@chem.uw.edu.pl (G.L.); 3 Department of Biology, Institute of Biology and Biotechnology, College of Natural Sciences, University of Rzeszow, Pigonia 1, 35-310 Rzeszow, Poland

**Keywords:** quercetin derivatives, senolytics, etoposide-induced senescence, breast cancer, inflammation

## Abstract

**Simple Summary:**

Cellular senescence may contribute to aging and age-related diseases, and the elimination of senescent cells is considered a promising anti-aging strategy. Drug-induced senescence in cancer cells during chemotherapy may also promote a number of adverse effects. Thus, in the present study, the usefulness of three quercetin derivatives as senolytic agents was studied upon stimulation of senescence program in breast cancer cells. We have shown that quercetin derivative with blocked hydroxy groups (QD3) sensitized etoposide-induced senescent breast cancer cells to apoptotic cell death that was accompanied by a decrease in proinflammatory and HSP70-based responses. We suggest that these prosenescent and senolytic activities can be combined to design a novel anti-cancer strategy, at least, against breast cancer cells.

**Abstract:**

Drug-induced senescence program may be activated both in normal and cancer cells as a consequence of chemotherapeutic treatment, leading to some adverse side effects such as senescence-associated secretory phenotype (SASP), secondary senescence, and cancer promotion. Targeted elimination of senescent cells can be achieved by drugs with senolytic activity (senolytics), for example, the plant-derived natural compound quercetin, especially when co-treated with kinase inhibitor dasatinib. In the present study, three quercetin derivatives were synthesized and tested for improved senolytic action against etoposide-induced senescent human normal mammary epithelial cells and triple-negative breast cancer cells in vitro. Transformation of catechol moiety into diphenylmethylene ketal and addition of three acetyl groups to the quercetin molecule (QD3 derivative) promoted the clearance of senescent cancer cells as judged by increased apoptosis compared to etoposide-treated cells. A QD3-mediated senolytic effect was accompanied by decreased SA-beta galactosidase activity and the levels of p27, IL-1β, IL-8, and HSP70 in cancer cells. Similar effects were not observed in senescent normal cells. In conclusion, a novel senolytic agent QD3 was described as acting against etoposide-induced senescent breast cancer cells in vitro. Thus, a new one-two punch anti-cancer strategy based on combined action of a pro-senescence anti-cancer drug and a senolytic agent is proposed.

## 1. Introduction

Cellular senescence is a heterogenous and complex stress response that can be triggered by endogenous factors and exogenous stimuli [1,2]. In general, senescent cells are characterized by permanent cell cycle arrest, proinflammatory secretory phenotype, and altered metabolic traits [3,4]. The induction of a senescence program may have both beneficial and detrimental effects such as tumor suppression, wound healing and tissue remodeling, and tumor promotion and aging [2,3,5]. It is widely accepted that the accumulation of senescent cells with age may contribute to the aging process and age-related diseases, and senescent cells may be considered a novel therapeutic target for anti-aging strategies [6,7].

To date, a number of compounds with senolytic activity (so called senolytics with the ability to preferentially kill senescent cells) have been reported, namely Bcl-2 family inhibitors, HSP90 inhibitors, p53 pathway targeting compounds, natural products and their analogs, cardiac glycosides, and galactose modified prodrugs [1,2,8,9]. Quercetin, a dietary flavonoid, was first documented to exert senolytic activity against senescent human endothelial cells and mouse bone marrow-derived mesenchymal stem cells (BM-MSCs) [10]. However, to show good and broad-spectrum senolytic action, quercetin should be co-administrated with a tyrosine kinase inhibitor dasatinib [10]. Other reported plant-derived natural substances or their analogs with senolytic activity are fisetin, piperlongumine and its analogs, EF-24 analog of curcumin, and GL-V9 analog of wogonin [11,12,13,14,15,16]. It has been postulated that synthetic analogs of natural substances may have more potent senolytic action compared to the corresponding unmodified compounds [8]. For example, the senolytic activity of curcumin may be limited because of its low bioavailability due to poor aqueous solubility and chemical instability due to keto-enol tautomerism [8]. Indeed, curcumin analog EF-24 was reported to be more effective than curcumin against different types of senescent cells that were executed by apoptosis induction and proteasomal degradation of Bcl-2 protein family members with anti-apoptotic functions [15]. Quercetin is also characterized by chemical instability, poor water solubility, and low bioavailability in biological systems, but these features can be overcome by using dedicated delivery systems such as lipid-based carriers, polymer nanoparticles, inclusion complexes, micelles, and conjugate-based delivery systems [17]. More recently, we have shown that quercetin surface-functionalized Fe_3_O_4_ nanoparticles promoted the elimination of oxidant-induced senescent human fibroblasts that was accompanied by increased activity of AMPK and diminished levels of proinflammatory factors such as IL-8 and IFN-β [18]. Thus, structural optimization is also needed to develop more potent senolytic agents such as synthetic analogs of quercetin and other natural substances. The increase of quercetin bioavailability can be also realized by modification of its structure in order to adjust the lipophilicity to specific target. The induction of tumor cell apoptosis by flavonoids includes their interactions with mitochondrial membranes, resulting in the collapse of their integrity (collapse of mitochondrial membrane potential). Some additional functionalities can be gained or removed due to site-specific protection/deprotection of hydroxy groups in flavonoids. For example, a direct antioxidant effect of flavonoids (due to scavenging of reactive oxygen species, ROS) can be turned into the pro-oxidant action in the presence of transition metal cations (in both processes a participation of free hydroxy groups is necessary) [19].

**Figure 1:**
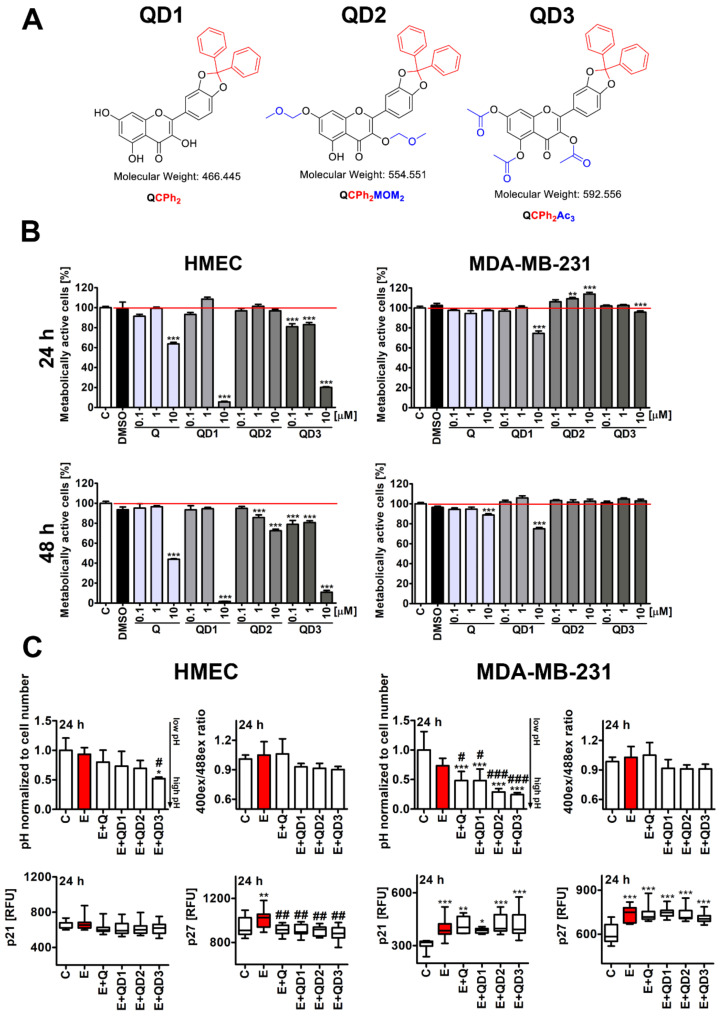
Short-term effects of quercetin derivatives QD1, QD2, and QD3 on selected viability and cell proliferation parameters in normal human mammary epithelial cells (HMEC cells) and MDA-MB-231 breast cancer cells upon stimulation with etoposide (E) at senescence-inducing concentration (1 µM). (**A**) The structures of 3,5,7-trihydroxy-2-(2,2-diphenylbenzo[d][1,3]dioxol-5-yl)-4H-chromen-4-one (QD1), 2-(2,2-diphenylbenzo[d][1,3]dioxol-5-yl)-5-hydroxy-3,7-bis(methoxymethoxy)-4H-chromen-4-one (QD2), and 3,5,7-triacetyloxy-2-(2,2-diphenylbenzo[d][1,3]dioxol-5-yl)-4H-chromen-4-one (QD3) are shown. (**B**) QD-mediated changes in metabolic activity. Cells were treated with Q or QDs at the concentrations of 0.1, 1, and 10 µM for 24 and 48 h, and metabolic activity was assayed using an MTT test. Metabolic activity at standard growth conditions (untreated samples) is considered 100%. To emphasize the action of Q or QDs, a red horizontal line is added. The solvent effect (DMSO) was also considered. Bars indicate SD, *n* = 6, *** *p* < 0.001, and ** *p* < 0.01 compared to control (C) (ANOVA and Dunnett’s a posteriori test). (**C**) QD-mediated changes in intracellular pH, GSH/GSSG redox potential, and the levels of cell cycle inhibitors p21 and p27 after 24 h treatment with etoposide (E, 1 µM, red) and 24 h post-treatment with Q or QDs (1 µM). Imaging cytometry and dedicated protocols were used. Intracellular alkalization was monitored as a decrease in fluorescent signals, and pH was normalized to cell number. Intracellular pH at control conditions is considered as 1.0. Immunosignals of p21 and p27 are presented as relative fluorescence units (RFU). GSH/GSSG redox potential was assessed using a Cellular Redox Sensor (roGFP-Grx1). RFU at two excitations (400 nm and 488 nm) was measured and GSH/GSSG redox potential is presented as a ratio of RFU_400nm_ to RFU_488nm_. Bars indicate SD or box and whisker plots are shown, *n* = 6, *** *p* < 0.001, ** *p* < 0.01, and * *p* < 0.05 compared to control (C) (ANOVA and Dunnett’s a posteriori test), ^###^ *p* < 0.001, ^##^ *p* < 0.01, ^#^ *p* < 0.05 compared to etoposide treatment (E) (ANOVA and Tukey’s a posteriori test).

In the present study, we synthesized three quercetin derivatives with catechol moiety converted into diphenylmethylene ketal (QD1, QD2, and QD3, Figure 1A), and differing with the number of remaining free hydroxy groups: in QD1, all three hydroxy groups are unblocked; in QD2, the OH at Positions 3 and 7 are protected with the methoxymethyl group; and derivative QD3 contains no free hydroxy groups because all are acetylated. Their senolytic action was then evaluated in comparison to quercetin-mediated senolysis in etoposide-induced senescent human normal mammary epithelial (HMEC) cells and MDA-MB-231 triple-negative breast cancer cells. We have previously established a chemotherapy-induced senescence model (1 µM etoposide treatment for 24 h) using four genetically different cancer cell lines, namely HeLa, MDA-MB-231, U-2 OS, and U-251 MG cells [20]. MDA-MB-231 breast cancer cells were considered as a cellular model of invasive breast cancer with limited drug responsiveness due to the lack of estrogen, progesterone, and HER2 receptors. In the present study, QD3 was documented as a novel senolytic agent as QD3 post-treatment potentiated apoptosis-based elimination of drug-induced senescent breast cancer cells that was accompanied by decreased secretory phenotype and HSP70 levels. Thus, a new protocol of combined chemotherapy and senotherapy as a one-two punch anti-cancer strategy was proposed that is effective at least against senescent triple-negative breast cancer cells in vitro.

## 2. Materials and Methods

### 2.1. Cell Lines and Drug-Induced Senescence Protocol

MDA-MB-231 human breast cancer cells (ATCC^®^ HTB-26^™^, ATCC, Manassas, VA, USA) and human mammary epithelial cells (HMEC, A10565, Thermo Fisher Scientific, Waltham, MA, USA) were used. MDA-MB-231 cells were maintained at 37 °C in a DMEM medium supplemented with 10% FBS and 100 U/mL penicillin, 0.1 mg/mL streptomycin, and 0.25 μg/mL amphotericin B (Corning, Tewksbury, MA, USA) in the presence of 5% CO_2_. HMEC cells were cultured in a HuMEC-ready medium (12752010, Thermo Fisher Scientific, Waltham, MA, USA) that consists of HuMEC basal serum-free medium (12753018) supplemented with HuMEC supplement kit (12755013, a mix of epidermal growth factor, hydrocortisone, isoproterenol, transferrin, insulin, and bovine pituitary extract). To stimulate drug-induced senescence, normal and cancer cells were treated with 1 µM etoposide (E1383, Merck KGaA, Darmstadt, Germany) for 24 h and then cultured without the drug for 7 days [20]. To study the senolytic activity of quercetin (Merck KGaA, Darmstadt, Germany) and its derivatives (QD1, QD2, and QD3, synthesized in the present study), 7 days after etoposide removal, cells were treated with quercetin and its derivatives at the concentration of 1 µM for 24 h. The concentration of 1 µM quercetin and its derivatives was selected on the basis of MTT assay. Moreover, for the analysis of selected parameters, cells were treated with 1 µM etoposide for 24 h and then post-treated with 1 µM quercetin or its derivatives for 24 h.

### 2.2. Synthesis of Quercetin Derivatives

Compound QD1 (3,5,7-trihydroxy-2-(2,2-diphenylbenzo[d][1,3]dioxol-5-yl)-4H-chromen-4-one) was prepared following the method proposed elsewhere [21,22] with some modifications. Diphenyldichloromethane (3.8 mL, 19.88 mmol) was added to a solution of quercetin (4.0 g, 13.24 mmol) in 120 mL of diphenyl ether. The mixture was stirred at 175 °C for 6 h, cooled to room temperature, and poured into cold petroleum ether. The precipitate was filtered and dissolved in ethyl acetate. Purification was performed by the means of silica column chromatography with DCM/MeOH (95:5) as an eluent and evaporated under the reduced pressure to give QD1 (3.80 g, 62%), as a yellow solid. Compound QD2 (2-(2,2-diphenylbenzo[d][1,3]dioxol-5-yl)-5-hydroxy-3,7-bis(methoxymethoxy)-4H-chromen-4-one) [23] was prepared using 1.0 g (2.15 mmol) of QD1 dissolved in 20 mL of dry acetone and was stirred with K_2_CO_3_ (1.22 g, 8.84 mmol) and chloromethyl methyl ether (0.80 mL, 10.53 mmol) at room temperature. The mixture was then refluxed gently for 24 h. After cooling to room temperature, the reaction mixture was filtered in order to remove K_2_CO_3_, and solvent was removed under reduced pressure. Purification of the residue was performed using silica column chromatography using DCM/MeOH (95:5) to give QD2 (0.30 g, 25%), as a yellow solid. To synthesize 3,5,7-triacetyloxy-2-(2,2-diphenylbenzo[d][1,3]dioxol-5-yl)-4H-chromen-4-one (QD3), the procedure was similar to proposed by Moine et al. and Cho et al. [22,24]. QD1 (2.0 g, 4.30 mmol) was dissolved in 11 mL of anhydrous pyridine, and after addition of acetic anhydride (2.4 mL, 25.00 mmol), the reaction mixture was stirred at room temperature for 20 h. Pyridine was removed by extraction (with three portions of 1 M HCl_aq_), the organic layer was washed with water, dried over Na_2_SO_4_, and filtered, and solvent was evaporated under reduced pressure. The crude product was purified by silica column chromatography using DCM/MeOH (98:2) to give QD3 (2.20 g, 87% yield) as a white powder. Nuclear magnetic resonance (NMR) spectroscopy was used to characterize the structure of three quercetin derivatives (^1^H NMR spectra can be found in the Supplementary Material).

### 2.3. Apoptosis and Necrosis

Apoptosis mediated by quercetin and its derivatives in etoposide-induced senescent normal and cancer cells (apoptosis-based senolytic activity) was studied using a Muse^®^ Cell Analyzer and Muse^®^ Annexin V and Dead Cell Assay Kit (phosphatidylserine externalization, Luminex Corporation, Austin, TX, USA), as previously described [20]. Necrosis mediated by quercetin and its derivatives in etoposide-induced senescent normal and cancer cells (necrosis-based senolytic activity) was revealed using a Muse^®^ Cell Analyzer and Muse^®^ Count and Viability Kit (Luminex Corporation, Austin, TX, USA) as previously described [25]. Viability profiles (subpopulations of live and membrane-damaged dead cells) were characterized using dedicated software.

### 2.4. Senescence-Associated β-Galactosidase Activity

Cellular senescence was assayed using CellEvent^™^ Senescence Green Detection Kit (Thermo Fisher Scientific, Waltham, MA, USA) as comprehensively described elsewhere [20].

### 2.5. Glutathione Redox Potential

Etoposide-, quercetin-, and quercetin derivatives-mediated changes in the glutathione redox potential (GSH/GSSG) were evaluated using a Premo^™^ Cellular Redox Sensor (roGFP-Grx1) (P36242, Thermo Fisher Scientific, Waltham, MA, USA) as comprehensively described elsewhere [26].

### 2.6. Intracellular pH

Intracellular pH was evaluated using pHrodo^™^ Green AM Intracellular pH Indicator (P35373, Thermo Fisher Scientific, Waltham, MA, USA) according to the manufacturer’s instructions. Briefly, live cells were stained with pHrodo^™^ Green AM reagent and fluorescent signals were measured using a Tecan Infinite^®^ M200 (Tecan Group Ltd., Männedorf, Switzerland) fluorescence mode microplate reader (λ_ex_ = 509, λ_em_ = 533). Intracellular alkalization was monitored as a decrease in fluorescent signals (relative florescence units, RFU) and normalized to cell number in a sample. Intracellular pH at control conditions is considered as 1.0.

### 2.7. Immunofluorescence

Cell fixation and immunostaining protocol was applied as previously reported [25]. The following primary and secondary antibodies were used, namely anti-p16 (1:200, MA5-17093), anti-p21 (1:800, MA5-14949), anti-p27 (1:200, PA5-27188), anti-IL-1β (1:200, P420B), anti-IL-8 (1:500, ab154390), anti-HSP70 (1:500, PA5-14521), and secondary antibodies conjugated to Texas Red (1:1000, T2767) or Alexa Fluor Plus 488 (1:1000, A32723) or Texas Red-X (1:1000, T6390) (Thermo Fisher Scientific, Waltham, MA, USA and Abcam, Cambridge, UK). Digital cell images were captured using a laser-based confocal imaging and HCA system IN Cell Analyzer 6500 HS (Cytiva, Marlborough, MA, USA). Quantitative analysis of protein levels as relative fluorescence units (RFU) was performed using IN Carta software (Cytiva, Marlborough, MA, USA).

### 2.8. Statistical Analysis

Data are presented as the mean ± SD from at least three independent experiments (cell cultures). In some experimental settings, box and whisker plots were also considered. Differences between control conditions and treated samples were evaluated using one-way ANOVA and Dunnett’s multiple comparison test, whereas differences between etoposide-treated cells and etoposide-treated and quercetin or quercetin derivative-post-treated cells were analyzed using one-way ANOVA and Tukey’s multiple comparison test. Statistical significance was revealed using GraphPad Prism 5. *p*-values of less than 0.05 were considered significant.

## 3. Results and Discussion

### 3.1. Short-Term Effects of Quercetin Derivatives against HMEC and MDA-MB-231 Cells

Except of relatively safe plant-derived natural substances with senolytic activity, other classes of senolytic compounds may promote on- and off-target toxicities that may potentially limit their clinical applications. For example, ABT-263 (navitoclax), a small-molecule Bcl-2 family protein inhibitor, may induce thrombocytopenia, while geldanamycin and 17-AAG, HSP90 inhibitors, may stimulate hepatotoxicity [27,28]. However, senolytic activity of natural substances such as curcumin and quercetin may be also limited due to poor water solubility, chemical instability, and low bioavailability [8,17].

Quercetin (3,3′,4′,5,6-pentahydroxyflavone, 2-(3,4-dihydroxyphenyl)-3,5,7-trihydroxy-4H-1-benzopyran-4-one) with non-protected hydroxy groups may undergo oxidative degradation (a half-life of quercetin is 10 h in PBS pH 7.4 and less than 30 min in cell culture media) [29,30]. The presence of five hydroxy groups makes this molecule highly polar, with limited mobility across the biomembranes, and susceptible to metabolic processes, decreasing the intracellular accumulation [30]. In order to minimize those drawbacks, three quercetin derivatives with selectively blocked hydroxy groups were designed and prepared, namely 3,5,7-trihydroxy-2-(2,2-diphenylbenzo[d][1,3]dioxol-5-yl)-4H-chromen-4-one (QD1), 2-(2,2-diphenylbenzo[d][1,3]dioxol-5-yl)-5-hydroxy-3,7-bis(methoxymethoxy)-4H-chromen-4-one (QD2), and 3,5,7-triacetyloxy-2-(2,2-diphenylbenzo[d][1,3]dioxol-5-yl)-4H-chromen-4-one (QD3) (Figure 1A and Figure S1). ^1^H NMR spectra of QD1, QD2, and QD3 are presented in Figures S2–S4, respectively.

First, short-term effects (24 and 48 h treatments) of three quercetin derivatives (QD1, QD2, and QD3) were compared with quercetin (Q) action at the concentrations of 0.1, 1, and 10 µM using two cellular models, namely human normal mammary epithelial (HMEC) cells and triple-negative MDA-MB-231 breast cancer cells (Figure 1B). In general, the metabolic activity of HMEC cells was more affected compared to the metabolic activity of MDA-MB-231 cells upon Q and QDs treatments (Figure 1B). As 10 µM QD1 and 10 µM QD3 treatments for 24 h promoted a massive decrease in metabolic activity of HMEC cells, this concentration was ruled out for further analysis (*p* < 0.001, Figure 1B). To study the senolytic effects of Q and QDs, a concentration of 1 µM was selected. We have previously established that 1 µM etoposide when treated for 24 h stimulated drug-induced senescence in four different cellular models of cancer [20]. Thus, to analyze QD-mediated senolytic effects in stress-induced senescent normal and cancer cells, the 1 µM etoposide (E) was used as a stimulator of cellular senescence.

We have studied short-term effects of 24 h treatment with E and a subsequent 24 h treatment with Q or QDs (Figure 1C). Except of QD3 post-treatment, E treatment, or Q or QD post-treatments did not affect intracellular pH in HMEC cells. In contrast, Q or QD post-treatments resulted in increased intracellular pH in MDA-MB-231 cells compared to untreated conditions (*p* < 0.001, Figure 1C). Q and QD post-treatments also accelerated this effect compared to E treatment (*p* < 0.001 and *p* < 0.05, Figure 1C). Q or QDs exerted limited effect on intracellular pH in HMEC or MDA-MB-231 cells (Figure S5). A total of 1 µM QD3 promoted a mild intracellular alkalization in HMEC cells (*p* < 0.05, Figure S5). A similar effect was not observed in MDA-MB-231 cells (Figure S5). It has been reported that 10 µM GL-V9 (5-hydroxy-8-methoxy-7-(4-(pyrrolidin-1-yl) butoxy)-4-H-chromen-4-one), a synthetic flavonoid derived from wogonin (5,7-dihydroxy-8-methoxy-2-phenyl-4*H*-1-benzopyran-4-one), induced apoptosis in senescent MDA-MB-231 cells that was achieved by lysosome alkalization and increased production of ROS [16]. Moreover, it has been postulated that the prooxidant activity of flavonoids may be important for their senolytic potential [31]. For example, fisetin, with higher senolytic activity than quercetin, has higher prooxidant effects than quercetin in the absence or presence of copper [31]. However, fisetin-based senolytic action is considered to be cell-specific. For example, fisetin is active against senescent human umbilical vein endothelial cells (HUVECs), but not against senescent IMR90 fibroblasts or primary human preadipocytes [11]. We have also consider to evaluate Q- or QD-mediated changes in redox homeostasis in normal and cancer cells (Figure 1C). Although E treatment or Q post-treatment slightly affected GSH/GSSG redox potential as judged by changes in redox sensor roGFP-Grx1 in both cell lines, this effect was not statistically significant (Figure 1C). QDs did not modulate GSH/GSSG redox potential (Figure 1C). Other natural substances or their analogs such as piperlongumine, an amide alkaloid isolated from long pepper and curcumin analog EF24, a synthetic analog of polyphenol derived from *Curcuma longa*, may induce apoptosis in senescent cells in a ROS production independent manner [13,15]. On the other hand, replacing the endocyclic C2–C3 olefin with an exocyclic methylene at C2 in the structure of piperlongumine yielded piperlongumine analogs 47–49 with increased senolytic activity that was accompanied by more potent production of ROS compared to unmodified piperlongumine [14]. Thus, the relationship between prooxidant activity and senolytic activity of natural products and their derivatives may be complex.

Short-term response in HMEC cells was also p21-independent (Figure 1C). In contrast, an increase in the levels of p21 was observed upon E treatment or Q or QD post-treatments in MDA-MB-231 cells that may suggest that all treatments resulted in p21-mediated inhibition of cell proliferation in breast cancer cells (Figure 1C). Similar effects were noticed in MDA-MB-231 cells in the case of changes in the levels of other cell cycle inhibitor, namely p27 (*p* < 0.001, Figure 1C). In HMEC cells, only E treatment promoted elevation in p27 levels (*p* < 0.01, Figure 1C). Except of Q- and QD1-mediated minor decrease in the levels of p21 in HMEC cells (*p* < 0.05, Figure S5), Q or QD alone treatment did not affect the levels of cell cycle inhibitors p21 and p27 in HMEC and MDA-MB-231 cells (Figure S5).

### 3.2. Quercetin Derivative QD3 Exerts Senolytic Activity against Etoposide-Induced Senescent Breast Cancer Cells

**Figure 2:**
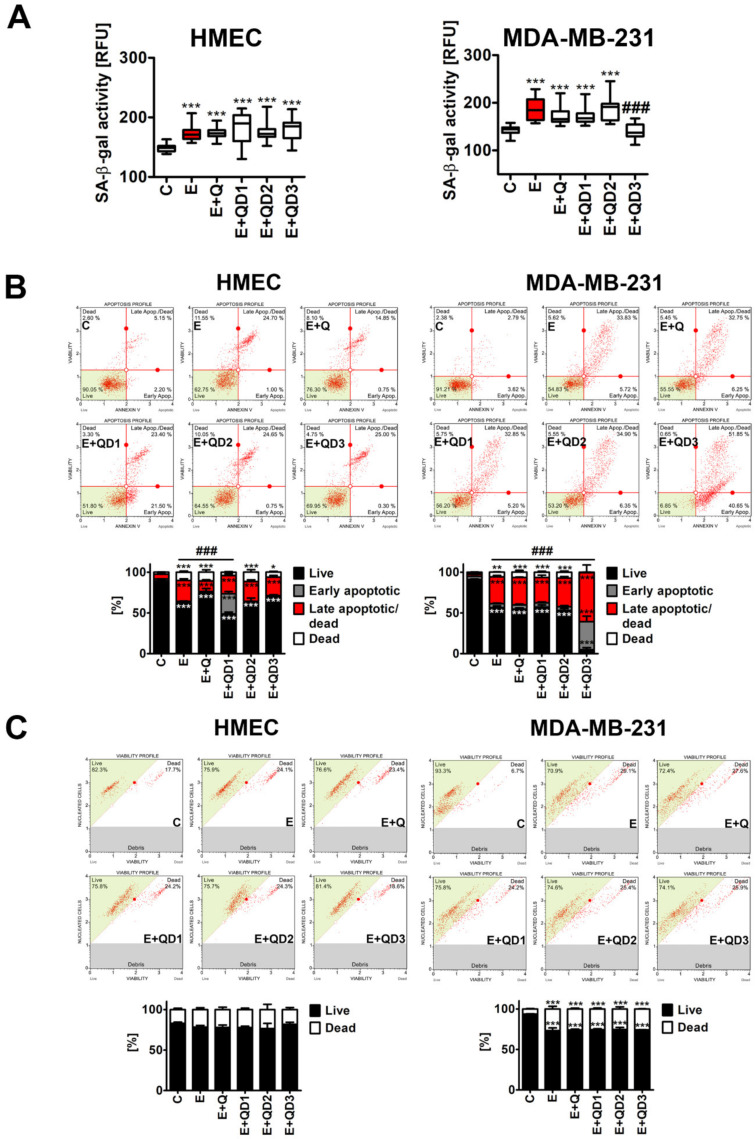
The effects of QDs on the levels of SA-beta-gal-positive cells (a senescence marker) (**A**) and apoptosis (**B**) and necrosis (**C**) induction in etoposide-induced senescent HMEC and MDA-MB-231 cells. Cells were treated with 1 µM etoposide (E) for 24 h, cultured for 7 days without a drug (induction of senescence program), and then treated with 1 µM Q or QDs for 24 h. (**A**) Cellular senescence was assayed using imaging cytometry and dedicated protocol. SA-beta-gal activity is presented as relative fluorescence units (RFU). Box and whisker plots are shown, *n* = 6, *** *p* < 0.001 compared to control (C) (ANOVA and Dunnett’s a posteriori test), ^###^ *p* < 0.001 compared to etoposide treatment (E, red) (ANOVA and Tukey’s a posteriori test). (**B**) Apoptosis-based senolysis was evaluated using flow cytometry and Annexin V staining. Bars indicate SD, *n* = 6, *** *p* < 0.001, ** *p* < 0.01, and * *p* < 0.05 compared to control (C) (ANOVA and Dunnett’s a posteriori test), ^###^ *p* < 0.001 compared to etoposide treatment (E) (ANOVA and Tukey’s a posteriori test). Representative dot plots are also shown. (**C**) Necrosis-based senolysis was evaluated using flow cytometry and dedicated protocol (dual-staining using a membrane-permeant DNA staining dye and a DNA-binding dye staining cells that have lost their membrane integrity). Bars indicate SD, *n* = 6, and *** *p* < 0.001 compared to control (C) (ANOVA and Dunnett’s a posteriori test). Representative dot plots are also shown.

Second, drug-induced senescence was promoted using 1 µM etoposide treatment for 24 h, and 7 days after drug removal, senescence-associated beta-galactosidase (SA-beta-gal) activity was monitored (Figure 2A).

Etoposide-mediated senescence was more pronounced in MDA-MB-231 breast cancer cells compared to HMEC normal cells (Figure 2A). An increase of 30% and 17% of SA-beta-gal-positive cells was observed in MDA-MB-231 and HMEC cells upon E treatment, respectively (*p* < 0.001, Figure 2A). A total of 1 µM Q or QDs post-treatments did not affect the levels of SA-beta-gal-positive cells compared to E treatment in HMEC cells (Figure 2A). In contrast, QD3 post-treatment caused a decrease in the levels of SA-beta-gal-positive cells compared to E treatment in MDA-MB-231 cells (*p* < 0.001, Figure 2A). Thus, one may speculate that QD3-associated diminution in senescent MDA-MB-231 cells may be mediated by senolytic activity of QD3. We have then compared the ability to promote apoptosis and necrosis by Q and QDs in both cell lines upon induction of the senescence program (Figure 2B,C). First, we ruled out the possibility that Q or QDs may be cytotoxic themselves, when used at the concentration of 1 µM for 24 h. No apoptotic and necrotic activity of Q or QDs was observed in HMEC and MDA-MB-231 cells (Figure S6). In contrast, apoptotic and/or necrotic modes of cell death were accompanied by E treatment or Q or QDs post-treatment in both cell lines (Figure 2B,C). However, only in MDA-MB-231 breast cancer cells, QD3 potentiated E-promoted apoptosis (*p* < 0.001, Figure 2B). QD3 promoted both early apoptotic events as well as late apoptotic events in senescent MDA-MB-231 breast cancer cells (*p* < 0.001, Figure 2B). As a similar effect was not observed in the case of necrotic cell death (Figure 2C), one can conclude that QD3-based elimination of senescent MDA-MB-231 breast cancer cells is mediated by apoptotic cell death (Figure 2B). In HMEC cells, QD1 post-treatment also promoted early apoptotic events compared to E treatment (*p* < 0.001, Figure 2B), but this effect was not accompanied by a decrease in the levels of senescent HMEC cells (SA-beta-gal-positive cells, Figure 2A). A total of 1 µM Q post-treatment did not potentiate etoposide-induced cell death in senescent HMEC and MDA-MB-231 cells (Figure 2). However, Q, when used at much higher concentrations, may exert some senolytic effects, but this action may be also considered cell-specific. For example, optimal Q concentrations for inducing death of senescent human preadipocytes and human umbilical vein cells were reported to be 20 and 10 µM, respectively [10]. Curcumin is also characterized as a relatively weak senolytic agent, while their analogs with improved bioavailability and biological efficiency, for example EF24, may exert more potent senolytic activity compared to unmodified curcumin [12,15].

### 3.3. Quercetin Derivative QD3-Mediated Decrease in the Levels of p27, IL-1β, IL-8 and HSP70 in Etoposide-Induced Senescent Breast Cancer Cells

**Figure 3:**
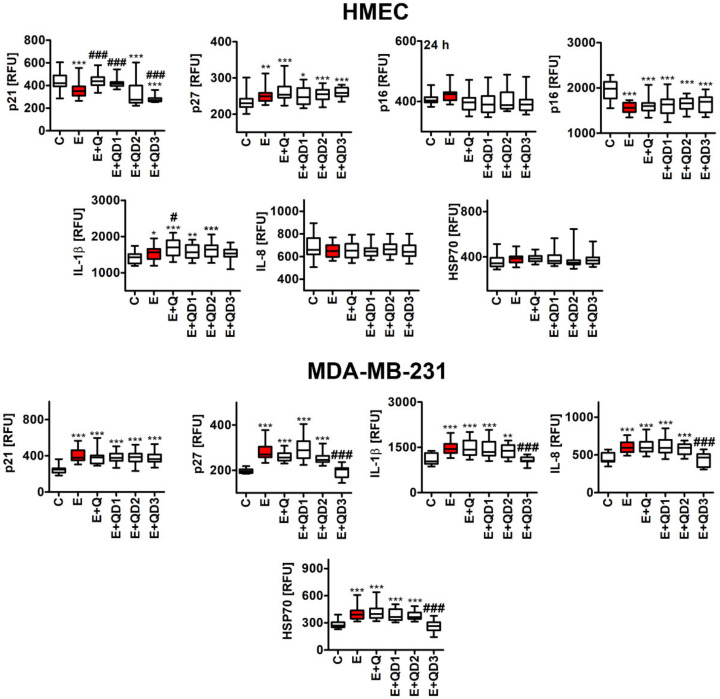
The effects of QDs on the levels of cell cycle inhibitors p21, p27, and p16 and proinflammatory factors IL-1β and IL-8 and stress sensor HSP70 in etoposide-induced senescent HMEC (**top**) and MDA-MB-231 (**bottom**) cells. Cells were treated with 1 µM etoposide (E, red) for 24 h, cultured for 7 days without a drug (induction of senescence program), and then treated with 1 µM Q or QDs for 24 h. The levels of p16 in HMEC cells were also assessed after 24 h treatment with E and 24 h post-treatment with Q or QDs. Immunosignals of p21, p27, p16, IL-1β, IL-8, and HSP70 were revealed using imaging cytometry and dedicated immunofluorescence protocol. Immunosignals of p21, p27, p16, IL-1β, IL-8, and HSP70 are presented as relative fluorescence units (RFU). Box and whisker plots are shown, *n* = 6, *** *p* < 0.001, ** *p* < 0.01, * *p* < 0.05 compared to control (C) (ANOVA and Dunnett’s a posteriori test), ^###^ *p* < 0.001, and ^#^ *p* < 0.05 compared to etoposide treatment (E) (ANOVA and Tukey’s a posteriori test).

We were also interested if all treatments may have permanent effects on the levels of cell cycle inhibitors, namely p21 and p27 in MDA-MB-231 breast cancer cells. Indeed, after 7 days of etoposide removal and Q, QD1, QD2, and QD3 post-treatments, the levels of p21 were elevated in senescent MDA-MB-231 cells (Figure 3).

Except of QD3, similar effects were observed in the case of the levels of p27 in senescent breast cancer cells (Figure 3). QD3 post-treatment resulted in decreased p27 levels compared to E treatment in senescent breast cancer cells (*p* < 0.001, Figure 3). Elevated levels of p27 were also observed in senescent HMEC cells after all treatments (Figure 3). In contrast, p21 levels were not increased in senescent HMEC cells (Figure 3). We have also considered the levels of other cell cycle inhibitors, namely p16, in HMEC cells after 24 h stimulation and during drug-induced senescence, but no augmentation in the pools of p16 was noticed in HMEC cells (Figure 3). As cellular senescence is characterized by senescence-associated secretory phenotype (SASP) [3,4], we have also evaluated the levels of selected proinflammatory factors such as IL-1β and IL-8 (Figure 3). Etoposide-induced senescence in MDA-MB-231 cells was accompanied by increased levels of IL-1β and IL-8 (Figure 3). In senescent HMEC cells, the levels of IL-1β were also increased, but the levels of IL-8 were unaffected (Figure 3). QD3 post-treatment in senescent MDA-MB-231 cells caused a decrease in the levels of IL-1β and IL-8 (Figure 3). Quercetin derivatives did not lower the interleukin levels in etoposide-induced senescent HMEC cells (Figure 3). Q or QDs also did not promote an inflammatory response upon alone treatment for 24 h in both cell lines (Figure S7). More recently, we have also reported that 5 μg/mL quercetin surface functionalized Fe_3_O_4_ nanoparticles suppressed senescence-associated proinflammatory response in oxidant-induced senescent human fibroblasts as judged by decreased levels of secreted IL-8 and IFN-β [18]. The senolytic activity of curcumin against human senescent intervertebral disc (IVD) cells was also accompanied by a curcumin-mediated decrease in proinflammatory markers that was mechanistically executed by the downregulation of Nrf2 and NF-κB pathways [32].

Selected HSP90 inhibitors were documented as a novel class of senolytics, as their cleared senescent cells and limited age-related symptoms in progeroid mice [33]. Similar to quercetin and Bcl-2 family inhibitors, HSP90 inhibitors promoted killing of senescent cells by the modulation of cell survival pathways, for example, by stimulating a decrease in AKT activity [10,33]. HSP70 proteins, molecular chaperones and multifunctional stress regulators, are ubiquitously presented in cancer cells and their levels are correlated with tumor survival and growth [34]. Thus, HSP70s may suppress multiple apoptotic and necrotic pathways, attenuate cellular senescence program, interfere with tumor immunity, and stimulate angiogenesis and metastasis, and HSP70 inhibitors may have anticancer function(s) [34]. Indeed, HSP70-2 was found be to overexpressed in a number of breast cancer patients and several breast cancer cell lines, for example, MDA-MB-231 cells [35]. Downregulation of HSP70-2 in MDA-MB-231 cells limited cell growth and motility and promoted cell cycle arrest and apoptosis [35]. HSP70 may bind and inhibit the activity of pro-apoptotic proteins in different types of cancer cells [36]. For example, HSP70 may attenuate etoposide-induced apoptosis in cancer cells by binding to caspase 3, thus inhibiting its pro-apoptotic properties [36]. HSP70 was also elevated in etoposide-induced senescent breast cancer cells and QD3 post-treatment resulted in decreased levels of HSP70 compared to E treatment (*p* < 0.001, Figure 3). Perhaps, etoposide disrupted proteostasis in breast cancer cells that promoted senescence program and HSP70-based adaptive stress response (Figure 3). In turn, QD3-mediated cytotoxicity against senescent breast cancer cells may be associated with QD3-based diminution in the levels of HSP70 (Figure 2B and Figure 3). Quercetin is a well-documented HSP70 inhibitor in different in vitro and in vivo models of cancer [37]. For example, quercetin attenuated HSP70 accumulation in tumors after combination therapy and induced apoptotic cell death via HSF1 pathway [38]. However, high concentrations of quercetin must be used to observe quercetin-mediated inhibition of HSP70 in breast cancer cells [39]. Quercetin, when used at the concentrations of 10, 25, and 100 µM, decreased the levels of HSP27, HSP70, and HSP90 that were accompanied by apoptotic cell death in MCF-7 and MDA-MB-231 breast cancer cells [39]. Taking into account the low bioavailability of quercetin, high concentrations of quercetin would be hard to achieve in vivo and thus this may limit its clinical applications based on quercetin-mediated HSP70 inhibition and apoptosis induction in cancer cells. In contrast, QD3, when used at relatively low concentration of 1 µM, caused a decrease in the levels of HSP70 in etoposide-induced senescent MDA-MB-231 breast cancer cells that sensitized them to apoptotic cells death (Figure 2B and Figure 3). Thus, structural optimization of quercetin may improve the bioavailability and biological efficiency of quercetin derivatives compared to unmodified quercetin (QD3, in this study). Other plant substances have been previously shown to interact with regulatory proteins to decrease viability of senescent cells [40]. For example, oxidation resistance 1 (OXR1) is elevated in senescent cells, and piperlongumine may bind to OXR1 and facilitate its degradation through the proteasome that may sensitize senescent cells to oxidative stress and perhaps contribute to cell death [40].

## 4. Conclusions

**Figure 4:**
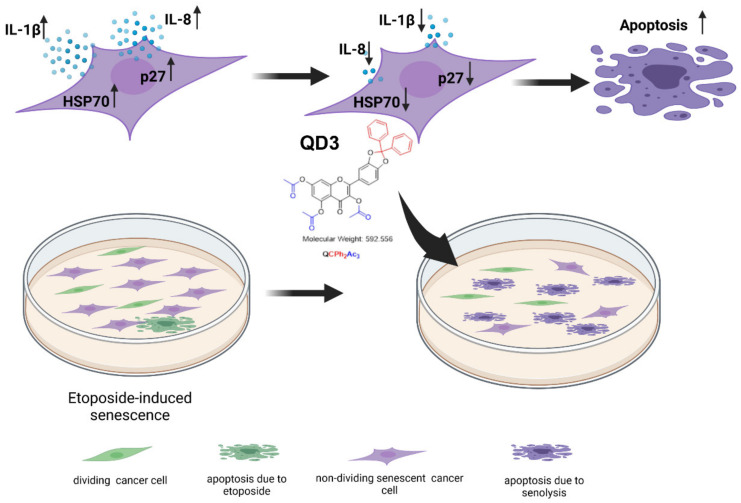
QD3 promotes apoptosis-based elimination of etoposide-induced senescent MDA-MB-231 breast cancer cells. Drug-induced senescence was characterized by elevated levels of cell cycle inhibitor p27, proinflammatory factors IL-1β and IL-8 (SASP biomarkers) and stress response protein HSP70 and QD3-mediated senolytic effect was accompanied by decreased pools of p27, IL-1β, IL-8, and HSP70. Mechanistically, QD3 may sensitize drug-induced senescent breast cancer cells to apoptotic cell death by lowering the levels of HSP70. ↑ indicates an increase in the levels of a biomarker and ↓ indicates a decrease in the levels of a biomarker.

In conclusion, we have documented for the first time that QD3, when used at a relatively low concentration of 1 µM, possessed senolytic activity in etoposide-induced senescent breast cancer cells that was accompanied by decreased pools of p27, IL-1β, IL-8, and HSP70 (Figure 4). Of course, more studies are needed to evaluate broad-spectrum senolytic activity of QD3, namely if QD3 may be also active against other types of drug-induced senescent cancer cells and may be considered as a part of one-two punch anticancer strategy to sensitize drug-resistant cancer cells and promote their elimination by senotherapy. Moreover, as etoposide promoted less pronounced senescence program in normal corresponding mammary epithelial cells compared to breast cancer cells (this study), more experimental approaches are needed to document senolytic action of QD3 also in other normal cells and propose its therapeutic applications.

## Data Availability

Additional data can be found in Supplementary Material.

## Supplementary Materials

The following are available online at https://www.mdpi.com/article/10.3390/cancers14030605/s1, Figure S1: Synthesis of quercetin derivatives QD1, QD2 and QD3 with selectively blocked hydroxy groups, Figure S2: ^1^H NMR spectrum of quercetin derivative QD1 (3,5,7-trihydroxy-2-(2,2-diphenylbenzo[d][1,3]dioxol-5-yl)-4H-chromen-4-one) in DMSO-d_6_, Figure S3: ^1^H NMR spectrum of quercetin derivative QD2 (2-(2,2-diphenylbenzo[d][1,3]dioxol-5-yl)-5-hydroxy-3,7-bis(methoxymethoxy)-4H-chromen-4-one) in CDCl_3_, Figure S4: ^1^H NMR spectrum of quercetin derivative QD3 (3,5,7-triacetyloxy-2-(2,2-diphenylbenzo[d][1,3]dioxol-5-yl)-4H-chromen-4-one) in CDCl_3_, Figure S5: Short-term effects of quercetin derivatives QD1, QD2 and QD3 on intracellular pH and the levels of cell cycle inhibitors p21 and p27 in normal human mammary epithelial cells and MDA-MB-231 breast cancer cells, Figure S6: The effects of QDs on apoptosis (A) and necrosis (B) induction in HMEC and MDA-MB-231 cells, Figure S7: The effects of QDs on the levels of proinflammatory cytokine IL-8 (HMEC, top and MDA-MB-231 cells, bottom) and IL-1β in MDA-MB-231 cells (bottom).
