# Supplementary material for: Senolysis-Based Elimination of Chemotherapy-Induced Senescent Breast Cancer Cells by Quercetin Derivative with Blocked Hydroxy Groups
Source: Cancers (Basel). 2022 Jan 25;14(3):605. doi: 10.3390/cancers14030605 (PMC8833762; doi:10.3390/cancers14030605)
Supplement: Supplementary file 1 [file cancers-14-00605-s001.zip › cancers-1514068-supplementary.pdf]

cancers

Supplementary Materials

Senolysis-Based Elimination of Chemotherapy-Induced Senescent Breast Cancer Cells by Quercetin Derivative with Blocked Hydroxy Groups

Anna Lewińska [1,*,†], Paweł Przybylski [2,†], Jagoda Adamczyk-Grochala [1], Dominika Błoniarz [1], Grzegorz Litwinienko [2,‡] and Maciej Wnuk [3,*,‡]

[1] Department of Biotechnology, Institute of Biology and Biotechnology, College of Natural Sciences, University of Rzeszow, Pigonia 1, 35-310 Rzeszow, Poland; jadamczyk@ur.edu.pl (J.A.-G.); dbloniarz@ur.edu.pl (D.B.)
[2] Faculty of Chemistry, University of Warsaw, Pasteura 1, 02-093 Warsaw, Poland; p.przybylski5@uw.edu.pl (P.P.); litwin@chem.uw.edu.pl (G.L.)
[3] Department of Biology, Institute of Biology and Biotechnology, College of Natural Sciences, University of Rzeszow, Pigonia 1, 35-310 Rzeszow, Poland
* Correspondence: alewinska@ur.edu.pl (A.L.); mwnuk@ur.edu.pl (M.W.)
† These authors contributed equally to this work.
‡ These authors contributed equally to this work.

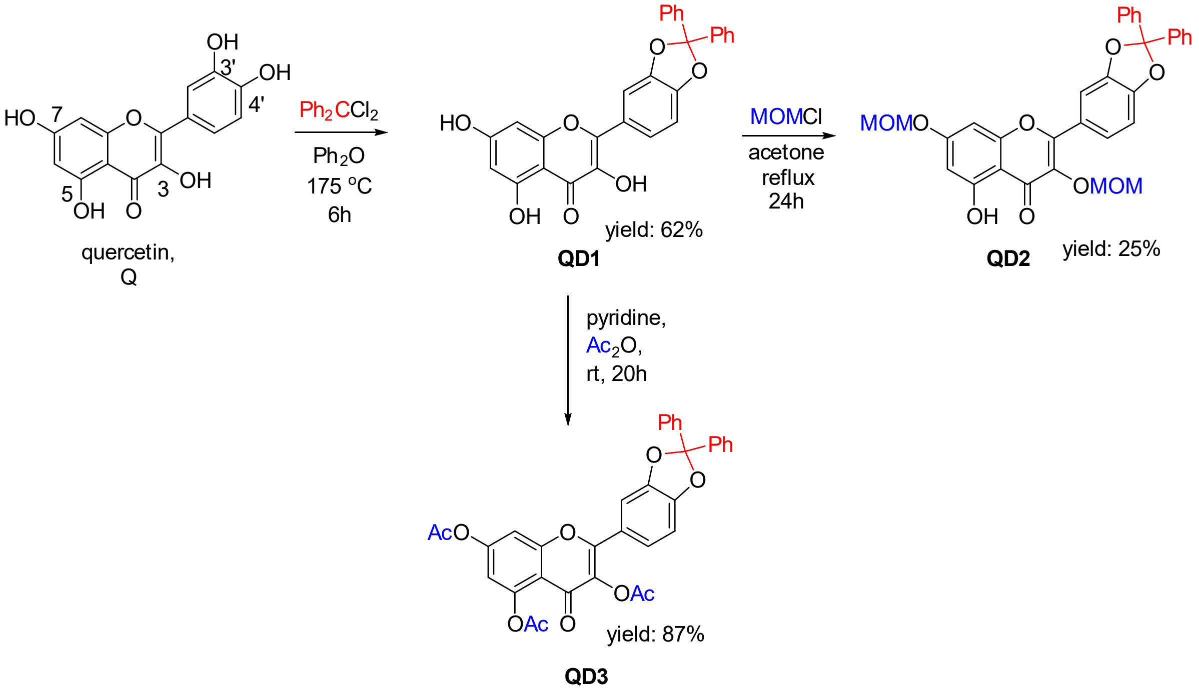

Figure S1. Synthesis of quercetin derivatives QD1, QD2 and QD3 with selectively blocked hydroxy groups. The numbering of the carbon atoms is presented in structure of Q.

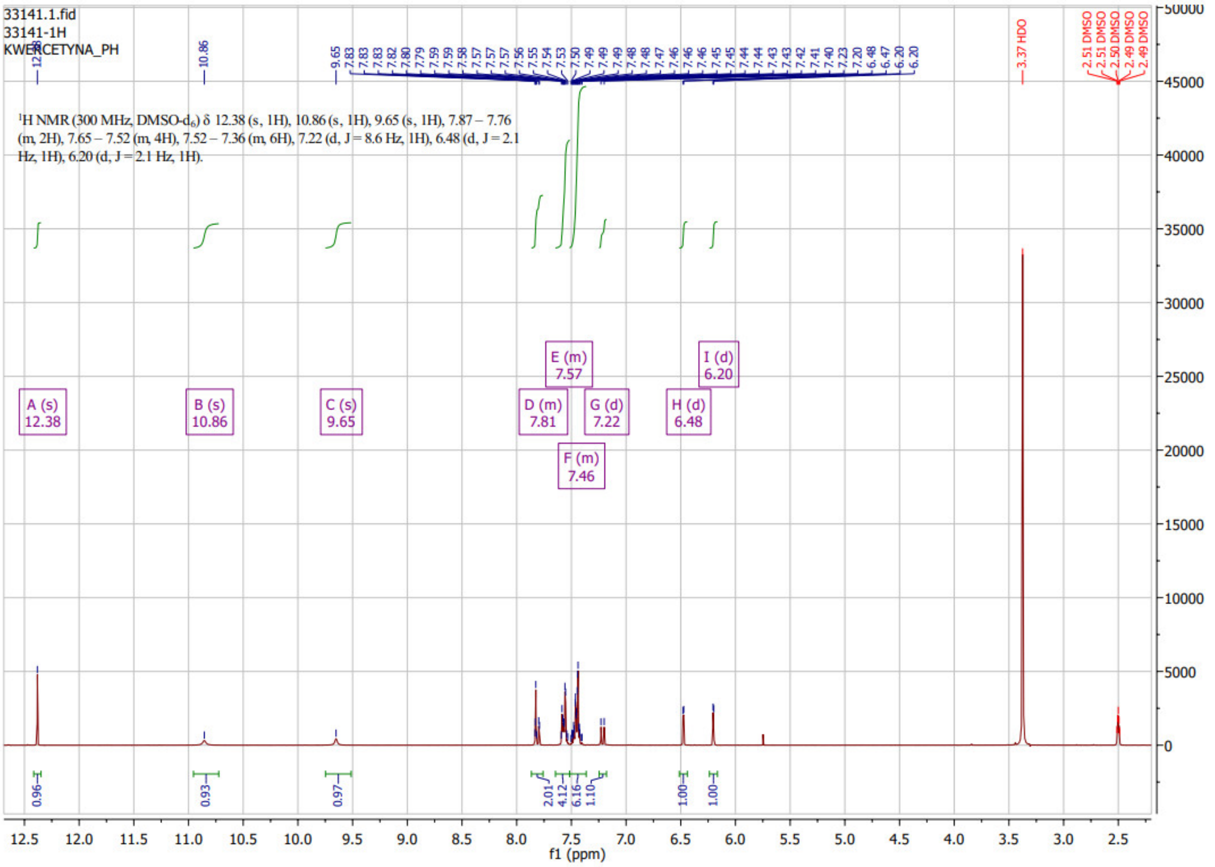

Figure S2. ^{1}H NMR spectrum of **quercetin derivative QD1 (3,5,7-trihydroxy-2-(2,2-diphenylbenzo[*d*][1,3]dioxol-5-yl)-4H-chromen-4-one)** in DMSO-d_6. (300 MHz, DMSO-d_6): δ 12.38 (s, 1H), 10.86 (brs, 1H), 9.65 (brs, 1H), 7.87 – 7.76 (m, 2H), 7.65 – 7.52 (m, 4H), 7.52 – 7.36 (m, 6H), 7.21 (d, J = 8.6 Hz, 1H), 6.48 (d, J = 2.1 Hz, 1H), 6.20 (d, J = 2.1 Hz, 1H).

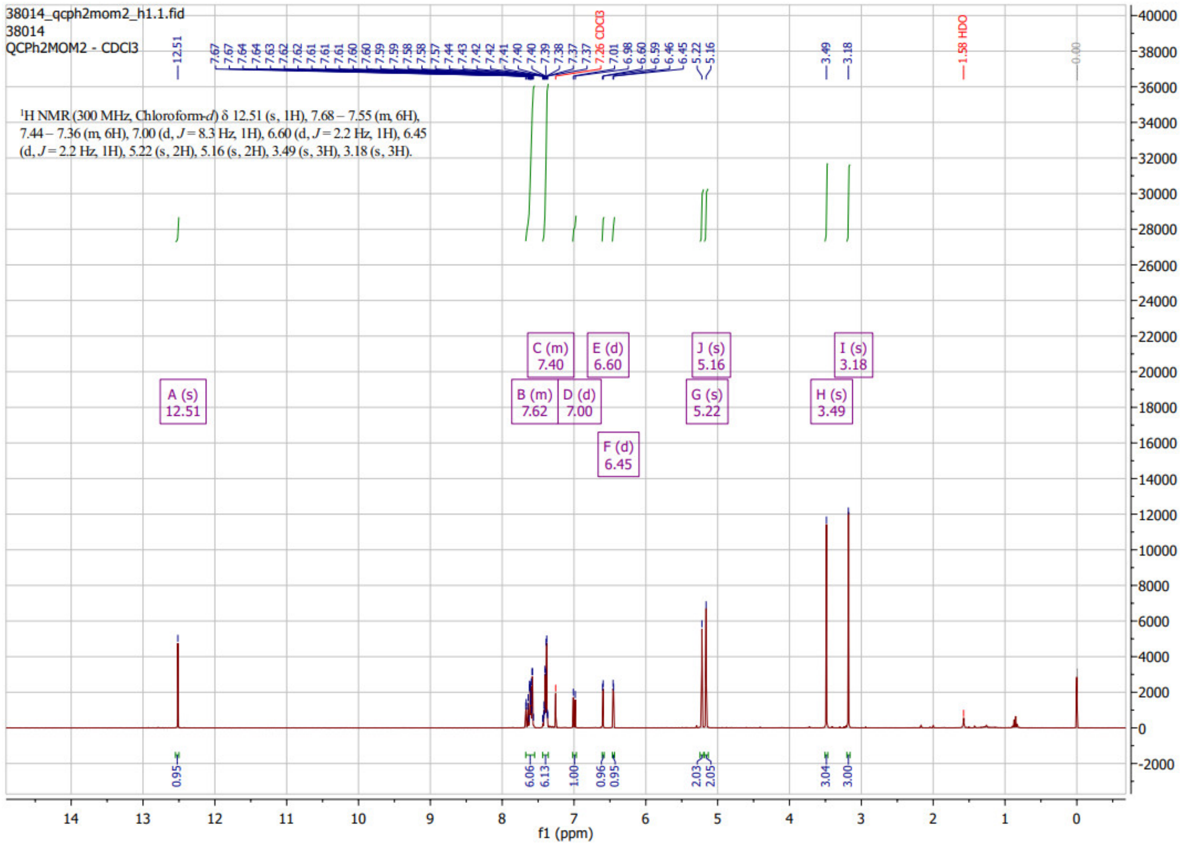

Figure S3. ^{1}H NMR spectrum of **quercetin derivative QD2 (2-(2,2-diphenylbenzo[*d*][1,3]dioxol-5-yl)-5-hydroxy-3,7-bis(methoxymethoxy)-4H-chromen-4-one)** in $CDCl_3$. (300 MHz, chloroform-*d*): δ 12.51 (s, 1H), 7.68 – 7.55 (m, 6H), 7.44 – 7.36 (m, 6H), 7.00 (d, J = 8.3 Hz, 1H), 6.60 (d, J = 2.2 Hz, 1H), 6.45 (d, J = 2.2 Hz, 1H), 5.22 (s, 2H), 5.16 (s, 2H), 3.49 (s, 3H), 3.18 (s, 3H).

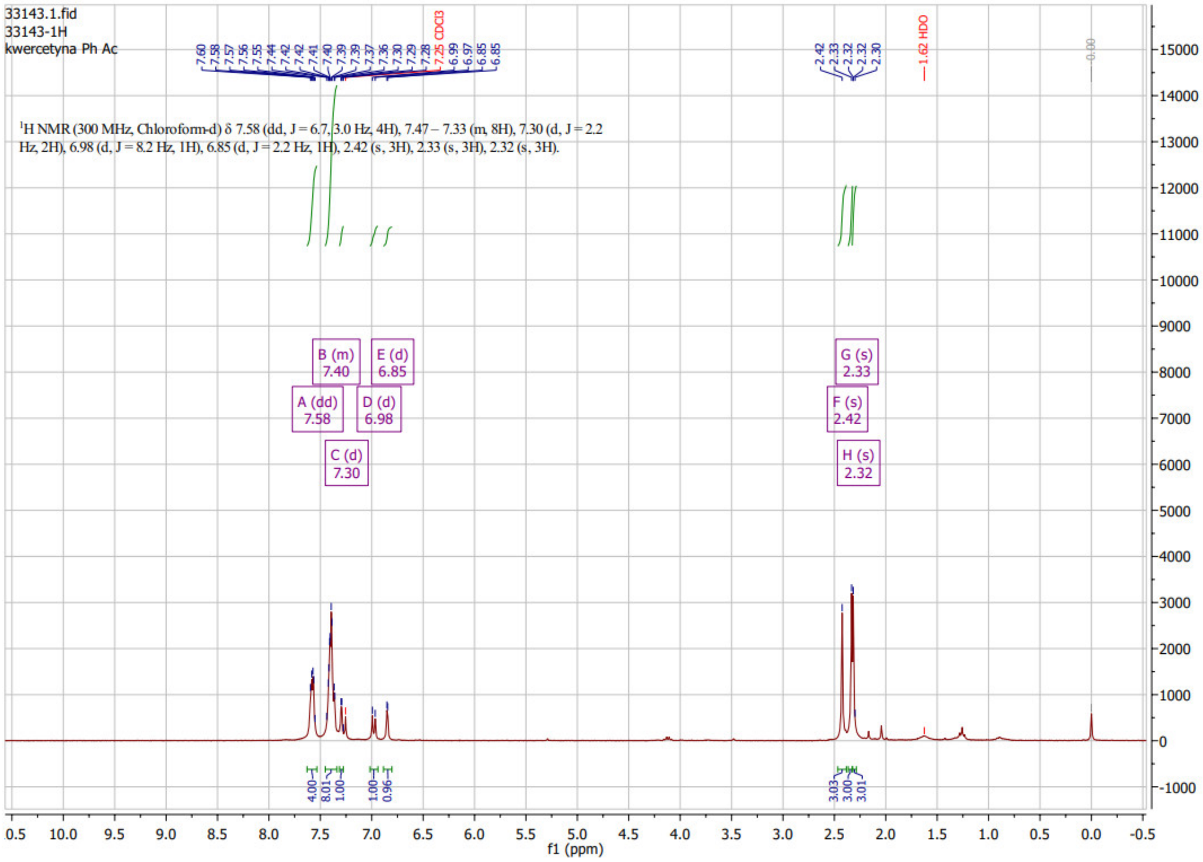

Figure S4. ^{1}H NMR spectrum of **quercetin derivative QD3 (3,5,7-triacetyloxy-2-(2,2-diphenylbenzo[*d*][1,3]dioxol-5-yl)-4H-chromen-4-one)** in $CDCl_3$. (300 MHz, chloroform-*d*): δ 7.58 (dd, J = 6.7, 3.0 Hz, 4H), 7.47 – 7.33 (m, 8H), 7.30 (d, J = 2.2 Hz, 1H), 6.98 (d, J = 8.2 Hz, 1H), 6.85 (d, J = 2.2 Hz, 1H), 2.42 (s, 3H), 2.33 (s, 3H), 2.32 (s, 3H).

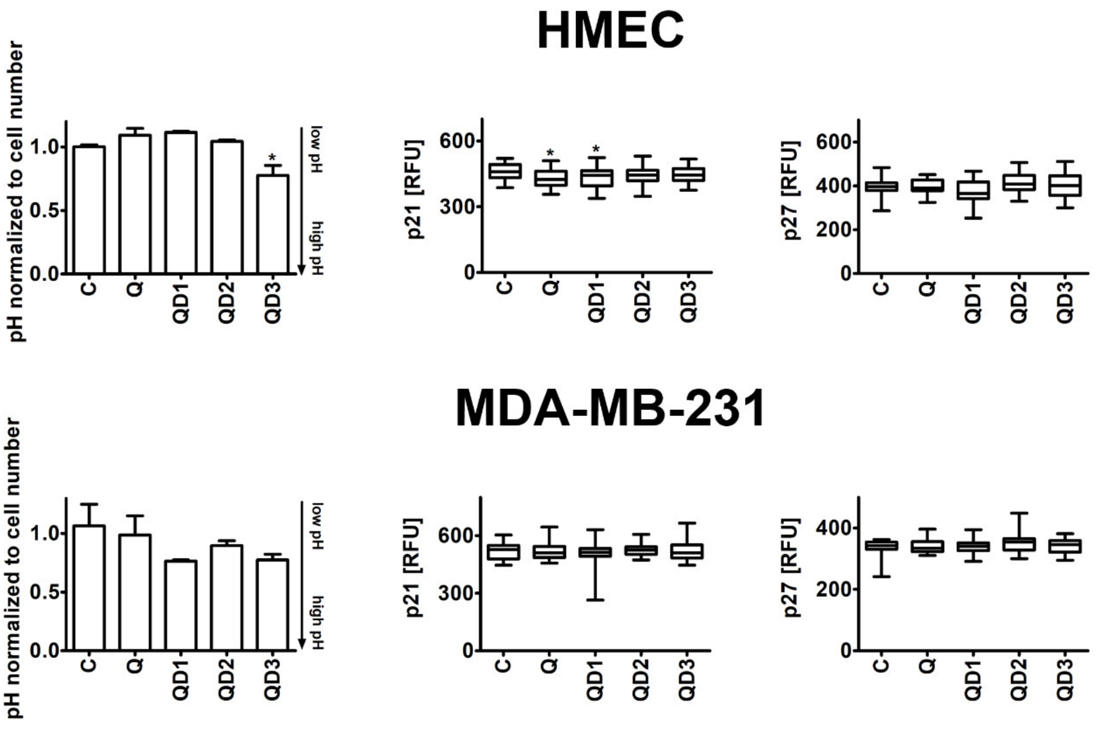

Figure S5. Short-term effects of quercetin derivatives QD1, QD2 and QD3 on intracellular pH and the levels of cell cycle inhibitors p21 and p27 in normal human mammary epithelial cells (HMEC cells, top) and MDA-MB-231 breast cancer cells (bottom). Cells were treated with Q or QDs at the concentration of 1 µM for 24 h. Imaging cytometry and dedicated protocols were used. Intracellular alkalization was monitored as a decrease in fluorescent signals and pH was normalized to cell number. Intracellular pH at control conditions is considered as 1.0. Immunosignals of p21 and p27 are presented as relative fluorescence units (RFU). Bars indicate SD or box and whisker plots are shown, n = 6, **p* < 0.05 compared to control (C) (ANOVA and Dunnett's a posteriori test).

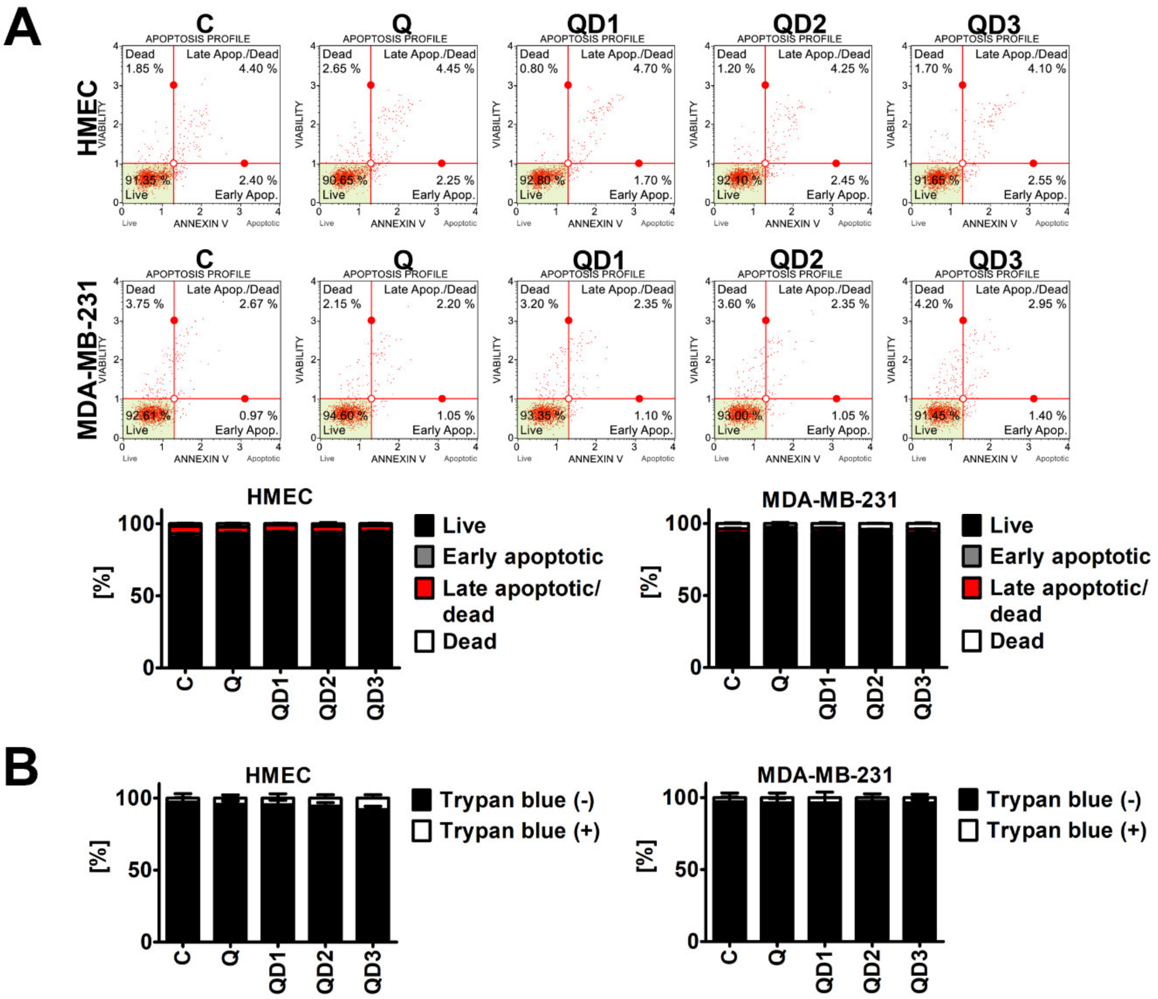

Figure S6. The effects of QDs on apoptosis (**A**) and necrosis (**B**) induction in HMEC and MDA-MB-231 cells. Cells were treated with 1 µM Q or QDs for 24 h. (**A**) Apoptosis was evaluated using flow cytometry and Annexin V staining. Bars indicate SD, n = 6. Representative dot plots are also shown. (**B**) Necrosis was evaluated using trypan blue staining. Bars indicate SD, n = 6. The percentage of live (non-stained) and dead (necrotic trypan blue-stained) cells is shown.

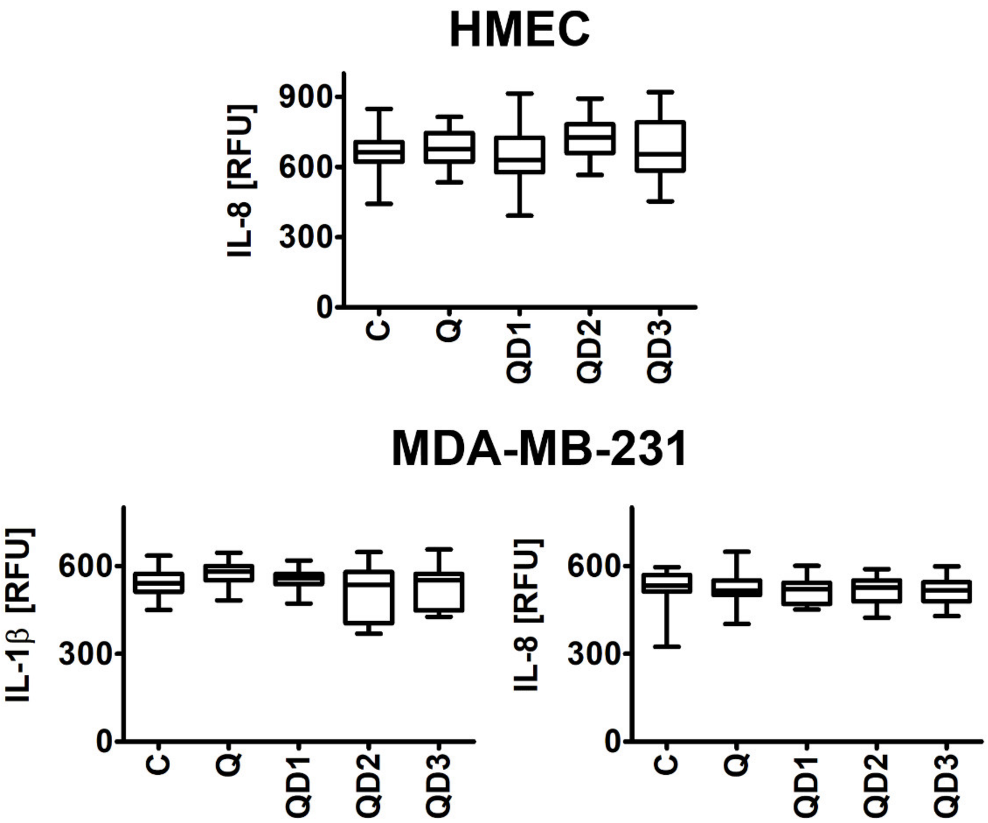

Figure S7. The effects of QDs on the levels of proinflammatory cytokine IL-8 (HMEC, top and MDA-MB-231 cells, bottom) and IL-1β in MDA-MB-231 cells (bottom). Cells were treated with 1 μM Q or QDs for 24 h. Immunosignals of IL-1β and IL-8 were revealed using imaging cytometry and dedicated immunofluorescence protocol. Immunosignals of IL-1β and IL-8 are presented as relative fluorescence units (RFU). Box and whisker plots are shown, n = 6.
